# Foliar Water Uptake of *Tamarix ramosissima* from an Atmosphere of High Humidity

**DOI:** 10.1155/2014/529308

**Published:** 2014-05-27

**Authors:** Shuang Li, Hong-lang Xiao, Liang Zhao, Mao-Xian Zhou, Fang Wang

**Affiliations:** ^1^Key Laboratory of Ecohydrology of Inland River Basin, Cold and Arid Regions Environmental and Engineering Research Institute, Chinese Academy of Sciences, Lanzhou 730000, China; ^2^University of Chinese Academy of Sciences, Beijing 100049, China

## Abstract

Many species have been found to be capable of foliar water uptake, but little research has focused on this in desert plants. *Tamarix ramosissima* was investigated to determine whether its leaves can directly absorb water from high humidity atmosphere and, if they can, to understand the magnitude and importance of foliar water uptake. Various techniques were adopted to demonstrate foliar water uptake under submergence or high atmospheric humidity. The mean increase in leaf water content after submergence was 29.38% and 20.93% for mature and tender leaves, respectively. In the chamber experiment, obvious reverse sap flow occurred when relative humidity (RH) was persistently above 90%. Reverse flow was recorded first in twigs, then in branches and stems. For the stem, the percentage of negative sap flow rate accounting for the maximum value of sap flow reached 10.71%, and its amount accounted for 7.54% of diurnal sap flow. Small rainfall can not only compensate water loss of plant by foliar uptake, but also suppress transpiration. Foliar uptake can appear in the daytime under certain rainfall events. High atmospheric humidity is beneficial for enhancing the water status of plants. Foliar uptake should be an important strategy of water acquisition for desert plants.

## 1. Introduction

Arid areas receiving ≤200 mm precipitation per year account for 26.6% of the total area of China [1]. Precipitation in extremely arid areas is several millimeters per year. Water shortage is one of the main climatic features in the arid region of Northwest China, and it is also one of the most important limitations to plant growth. According to statistics, mean annual precipitation in the lower reaches of the Heihe River is only 39 mm, while evaporation reaches 3534 mm. In such conditions, it is hard for desert plants to survive and reproduce if they rely entirely on precipitation, especially in this region with deep groundwater table. Thus, desert plants should have water utilization strategies adapted to drought environments. Previous research has shown that leaves of the desert plant* Bassia dasyphylla* improve leaf water status by dew absorption [2]. Many species with epidermal hydathodes of* Crassula* from the Namib Desert in southern Africa can benefit directly from dew or fog deposited on leaf surfaces [3].

The phenomenon of foliar uptake was studied as early as 1676 by Mariotte [4], and this got more attention gradually since the 20th century. It was proved that many species have the capacity to absorb rain, dew, or fog water directly into their leaves [5–9]. Plenty of evidence on foliar uptake has come from the coastal redwood ecosystem of California where the climate is temperate and Mediterranean with a long summer drought, and leaf-wetting events by fog frequently occur during drought season [10–13]; 80% of the dominant species in this ecosystem exhibit this foliar uptake water acquisition strategy [12]. Plants inhabiting subtropical and tropical forest ecosystems also exhibit foliar water uptake [9, 14, 15]. Leaves of epiphytes and non-epiphytes in Xishuangbanna have been shown to absorb fog water, even when not suffering from serious water stress [16]. Foliar water uptake is likely a common water acquisition mechanism for terrestrial plants.

Water absorbed through leaves not only refills the storage capacity in leaves, but also allows remaining water to pass through twigs, branches, and stems towards the belowground components [11, 15, 17, 18]. Moisture movement in the soil-plant-atmosphere continuum (SPAC) system is not unidirectional from soil through plants to the atmosphere but bidirectional [19]; water can flow equally well both ways through plants when a proper water potential gradient is established [20]. Previous research has shown that water can diffuse into leaves through the cuticle [9, 13], epidermal hydathodes [3], or absorbent trichomes [2, 21]. At present, study on the driving forces for, and processes of, absorption and transport of foliar water acquisition is relatively weak. In addition, the physiological and ecological responses to foliar water uptake have aroused concerns. Foliar uptake plays roles in preventing plants from dehydration [11], increasing water potential of leaves and branches [9, 22], enhancing photosynthetic rate [10, 23], and promoting plant growth [15]. However, the effect of foliar uptake on physiological processes is probably quite variable because of different cloud immersion patterns; for example, carbon gain is reduced because of leaf wetting by heavy fog in the daytime [24]. So far, little research has been conducted on foliar uptake of desert plants.

This study chose* Tamarix ramosissima* as the research object; this species is mainly distributed in arid-desert areas of Northwest China and has been widely used in sand fixation projects as well as in soil and water conservation. It was hypothesized that foliar water uptake will occur under high atmospheric humidity, and that this is an important water acquisition mechanism for* T. ramosissima* to improve plant water status and enhance physiological processes. A suite of experiments were conducted using a combination of sap flow, water potential, and ecophysiological measurements under field conditions to investigate the beneficial micrometeorological conditions for foliar uptake. In addition, this study provides information on the driving forces and pathways in the process of foliar water uptake. Results from this study demonstrate that water absorbed by foliage is important to the survival of desert plants and provide further knowledge on drought-resistance mechanisms for desert plants to accommodate an extremely arid environment.

## 2. Materials and Methods

### 2.1. Field Site Description

The research area is located in the transition zone between the Loess Plateau and the Tengger Desert in northwestern China, specifically in Sitan Village of Jingtai County, Gansu Province (37°14′N, 103°48′E, 1828 m a.s.l.). The temperate continent-arid climate of the site has a mean annual temperature of 8.6°C with extreme low and high temperatures of about −27.3°C in January and 38.6°C in July, respectively. The annual active accumulated temperature of ≥0 is ~3614.8°C; the frost-free period is ~120 d. The mean annual precipitation is ~180 mm, with uneven intra-annual distribution and low rainfall intensity; ~60% of the total rainfall falls from July to September, and winter and spring are very dry. The potential evaporation is 3038 mm year^−1^. The wind speed averages 2.0–3.1 m s^−1^, with an instantaneous maximum wind speed of 21.7 m s^−1^; blowing sand occurs frequently. Soil types are mainly sierozem and gray-brown desert soil. Since 1990, the local government has responded to the national policy to convert farmland to forest to protect the fragile environment.

In 2003, the study site was converted from farmland to forest consisting of the shrub* T. ramosissima* with herbaceous plants such as* Suaeda glauca* (Bunge) Bunge and* Chenopodium album* L. The plants of* T. ramosissima* were planted in a uniform pattern with 2 m spacing and 4 m between the rows. Mean tree height of* T. ramosissima* was 170 cm; mean basal diameter was 3.2 cm. No groundwater is available at the site. Water sources for plants mainly include precipitation and residual soil water, without irrigation.

### 2.2. Foliar Water Uptake Capacity Experiment

To measure the capacity for foliar water uptake of* T. ramosissima* and to recognize differences in leaf water content and foliar uptake between tender leaves and mature leaves, twigs were collected from several plants of* T. ramosissima* and roughly divided into two groups: the middle part of the canopy with dark-green mature leaves accounting for absolute proportion and the upper part of the canopy with tender leaves accounting for a higher proportion. The cut surface of each twig was immediately sealed with petroleum jelly to prevent evaporation.

The capacity for foliar water uptake of* T. ramosissima* was evaluated according to the method by Limm et al. [12]. The starting mass (Mass_1_, g) of the twig was measured rapidly with an electronic balance (0.001 g resolution) under the circumstance of wind-resistant shelter and then it was immediately submerged in deionized water with the cut end above the water line to prevent water entry. Leaves were submerged in darkness for 6 h to test potential foliar water uptake. Following rehydration, the twig was drawn out from the water and thoroughly patted dry with paper towels, and the mass was recorded again (Mass_2_, g). To avoid any residual water persisting on the leaf surface, the twig was allowed to air-dry for 10 s and it was weighed again (Mass_3_, g) and then submerged again in water for about 1 s, towel-dried, and reweighed (Mass_4_, g). Because leaves of* T. ramosissima* are short, bulbous, and scaly, calculating the leaf area is difficult. Therefore, leaves were removed from the detached twig when the fourth weight was recorded and the twig was weighed without leaves (Mass_5_, g). It was supposed that twigs without leaves cannot absorb water directly. Leaves were first dried at 105°C for 0.5 h and then dried at 80°C for 24 h and the dry weight was measured (*W*_Dry_, g). Six repetitive samples were set for mature leaves and tender leaves. The foliar uptake was standardized per dry weight (g).

The initial weight (*W*_I_, g) and the final weight (*W*_F_, g) of leaves were calculated by (1) and (2), respectively:

(1) $$\begin{array}{c} W_{\text{I}}={\text{Mass}}_{1}-{\text{Mass}}_{5}, \end{array}$$

(2) $$\begin{array}{c} W_{\text{F}}=\left({\text{Mass}}_{2}-{\text{Mass}}_{4}+{\text{Mass}}_{3}\right)-{\text{Mass}}_{5}. \end{array}$$

The initial leaf water content (LWC_I_, %) and the final (LWC_F_, %) were calculated by (3) and (4), respectively:

(3) $$\begin{array}{c} {\text{LWC}}_{\text{I}}=\frac{W_{\text{I}}-W_{\text{Dry}}}{W_{\text{I}}}\times 100, \end{array}$$

(4) $$\begin{array}{c} {\text{LWC}}_{\text{F}}=\frac{W_{\text{F}}-W_{\text{Dry}}}{W_{\text{F}}}\times 100. \end{array}$$

The amount of foliar water uptake (Uptake, g g^−1^) was calculated by evaluating the change in leaf water content before and after submergence as

(5) $$\begin{array}{c} \text{Uptake}=\left({\text{Mass}}_{2}-{\text{Mass}}_{1}\right)-\left({\text{Mass}}_{4}-{\text{Mass}}_{3}\right). \end{array}$$

The increased percentage of leaf water content (Rw, %) was expressed by

(6) $$\begin{array}{c} \text{Rw}=\frac{W_{\text{F}}-W_{\text{I}}}{W_{\text{I}}-W_{\text{Dry}}}\times 100. \end{array}$$

A one-sample *t*-test (*α* = 0.05) was used to determine if the quantity of foliar water uptake and the increase in leaf water content were significantly > zero and an ANOVA was used to examine differences between mature leaves and tender leaves.

### 2.3. High Air Humidity Exposure Experiment

Field experiments were conducted in an area dominated by* T. ramosissima* from July 17th to 29th, 2013. A 100 × 100 m (1 ha) representative plot was selected and all vegetation and sap flow measurements were conducted inside this plot. A single big* T. ramosissima* plant was selected; it had multiple branches at the base; its crown width was 2.95 ± 0.05 m in the east-west direction and 3.25 ± 0.05 m in the north-south direction. This* T. ramosissima* plant was roughly divided into two parts: one exposed to natural conditions as the control group and the other exposed to a controlled humidity chamber as the treatment group. The enclosed chamber was 3 × 1.8 × 1.8 m, and the joints between the plexiglass sheets were sealed by adhesive tape. The ceiling was covered between 6 and 7 pm every day and reopened between 6 and 7 am the next day. The humidifying experiment was suspended and the ceiling uncovered at night if consistent rain occurred during the day. A small* T. ramosissima* was selected and completely covered using a 1.2 × 1.2 × 1.2 m chamber; the operating procedure was the same as that for the large plant. Two ultrasonic humidifiers (Yadu Electronics, Beijing, China) were used to increase atmospheric moisture in the big chamber and one for the small chamber. This device produced water aerosol with 1–3 *μ*m droplets. On the opposite side of the chamber from the ultrasonic humidifier, a thermohygrograph (MicroLog PRO-EC750, Fourier Systems Ltd., Israel) monitored air temperature and relative humidity (RH) every 5 min. Calibration of the temperature/humidity measured by the thermohygrograph had been previously checked with the data measured by an automatic weather station (AWS; Type WS01, Delta-T, Cambridge, UK). The big chamber was humidified with two different water sources (drinking water and “labeled” water) (an oxygen isotopic composition (*δ* ^18^ *O*) of +50‰). Sap flow and water potential were measured during the experimental period.

#### 2.3.1. Sap Flow Measurement

To characterize foliar uptake in situ, the sap flow was continuously measured in* T. ramosissima* plants using sap flow gauges (Flow32, Dynamax Inc., Houston, TX, USA) with the energy balance principle. The heat balance method is very effective for measuring low and reverse rates of flow [25]. For the treatment group, a gauge was attached to the base of straight stem at least 40 cm above the soil surface, one for a lateral branch and one for a current growth branch in the stem. The stems with the attached gauges did not face the mist from the humidifier directly. Similarly, for the control group, three gauges were attached to a stem, branch, and twig. In addition, two gauges were attached to stems in the small chamber. Models SGA9, SGA10, SGA13, SGB19, and SGB25 were used to monitor variation in sap flow of different diameter stems of* T. ramosissima* during the entire field experiment.

Gauges were carefully installed following the manufacturer's instructions. Each gauge was wrapped with multiple layers of bubble foil insulation to shield it from rain and direct sunlight and reduce interference from ambient temperatures. Also, shelters were made and sealed with neutral silicate glass cement just above the gauges to prevent water flowing down the stems into the gauges. The outputs from the gauges were monitored every 10 s with programmable data loggers (CR 1000, Campbell Scientific, Logan, UT, USA). The measured signals were averaged every 1 min and recorded at 6 minute intervals.

#### 2.3.2. Water Potential

The water potential of leaves and shoots was measured using the PSYPRO water potential system (WESCOR, Inc.) before, during, and after the humidifying process every day during the field experiment; the water potential from the control group was also measured at the same time. Wetted leaf surfaces were carefully dried just prior to measurement. In addition, for the control group and treatment group, the changes of water content in leaves and stems were measured at the same time.

#### 2.3.3. Meteorological Measurements

In 2012, an automatic weather station (AWS; Type WS01, Delta-T) was set up ~200 m away from the experimental field. Meteorological data were recorded by a data logger at half-hour intervals. The meteorological factors included wind speed (m s^−1^), rainfall (mm), air relative humidity (RH, %), air temperature (*T*_*a*_, °C), soil temperature (°C), atmospheric pressure (hPa), net solar radiation (kW m^−2^), and photosynthetically active radiation (PAR, mmol). Vapor pressure deficit (VPD, hPa) was calculated based on RH and *T*_*a*_ measurements.

### 2.4. Data Analysis

All statistical analyses were performed with the SPSS software package (version 17.0 for windows, SPSS Inc., USA) with *α* = 0.05 as the threshold for statistical significance. Note that only data from the big chamber were analyzed; data from the small chamber are not shown in this study.

## 3. Results

### 3.1. Foliar Uptake Capacity

**Figure 1:**
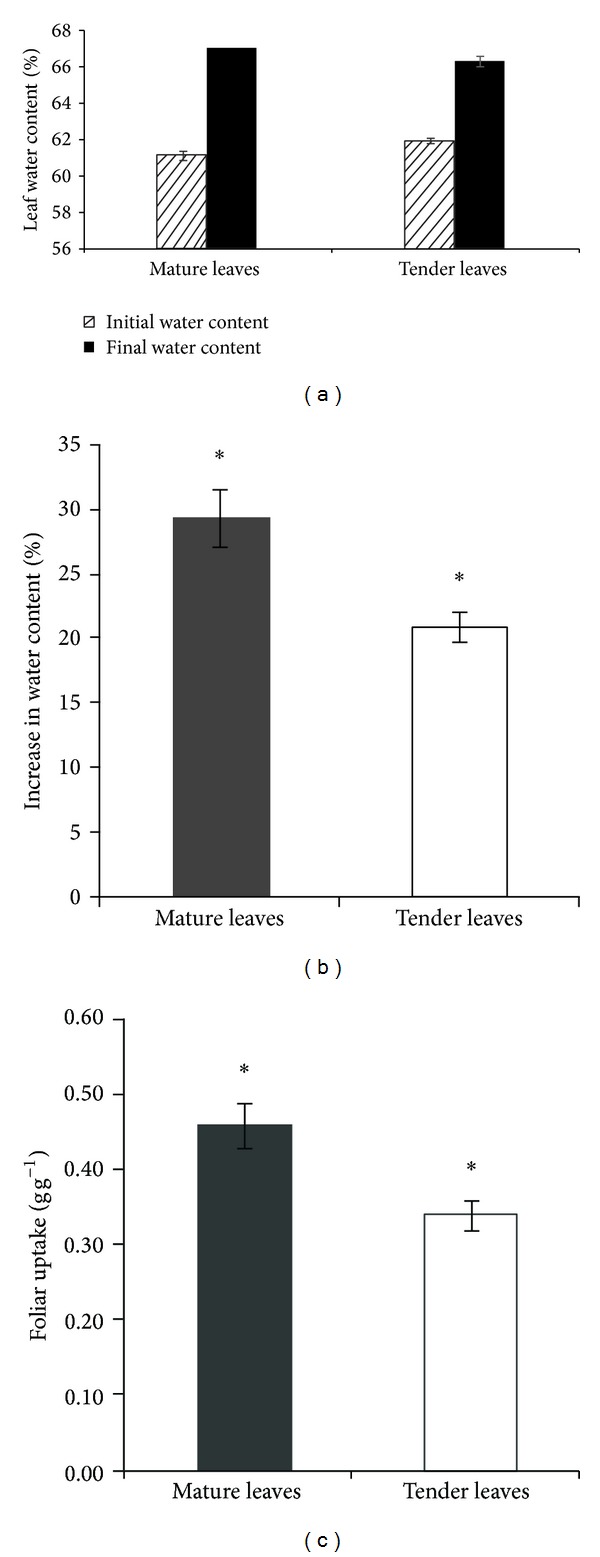
Water absorption by mature and tender leaves from detached twigs of* T. ramosissima* during submergence in deionized water for 6 h (*n* = 6). (a) The mean (±SE) leaf water content before and after submergence. (b) The mean (±SE) increase in leaf water content. (c) The mean (±SE) foliar uptake quantity standardized by leaf dry weight. The* asterisk* indicates increase in leaf water content or foliar uptake of the two leaf types reached significant level.

Both mature and tender leaves of* T. ramosissima* demonstrated a capacity for foliar water uptake during the 6 h submergence. Whether mature or tender, compared with that before submergence, the leaf water content increased significantly by the end of submergence (Figure 1(a)). Tender leaves were slightly higher in water content than mature leaves for the initial values but the mature leaves were slightly higher than tender leaves in final leaf water content. The two leaf types showed a significant increase in percentage of leaf water content (*P* < 0.05) with mature leaves having a 29.38% increase and tender leaves having a 20.93% increase (Figure 1(b)). Both of them exhibited significant (*P* < 0.05) water absorption per leaf dry weight, ~0.462 g g^−1^ for mature leaves and 0.341 g g^−1^ for tender leaves (Figure 1(c)). Mature leaves had significantly greater absorption and increase in the percentage of leaf water content than tender leaves (*F* = 34.47, *P* < 0.0001; *F* = 46.05, *P* < 0.0001, resp.).

### 3.2. Sap Flow

**Figure 2:**
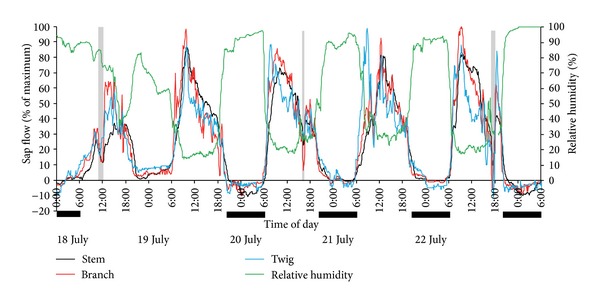
Five days of sap flow at the three different positions on the* T. ramosissima* plant under different air humidity conditions. Black bars represent that plant was exposed to high atmospheric humidity caused by the humidifier. Light gray bars represent precipitation events and the detailed information was provided in Table 1.

Figure 2 shows sap flow and air RH conditions over a 5-day period in the chamber in late July. Experiments on the sap flow of* T. ramosissima* exposed to high atmospheric humidity in the field were also conducted in August 2012 and September 2013. The sap flow patterns displayed in Figure 2 were similar to patterns during those other two periods. The sap flow data in Figure 2 were collected from different branches of the same stem. For ease of comparison, the differing flow rates in the different branch levels were ignored and values were normalized by expressing flow rates as a percentage of maximum values for each sensor in this measurement period. This permits a clear comparison of the timing and relative proportions of positive and reverse sap flow at each measurement position within the tree [11]. Three short and minimal rainfall events occurred during the 5 days (Table 1).

Sap flow of* T. ramosissima* had a significantly negative correlation with RH (Figure 2), and the correlation coefficient was −0.88 (*P* < 0.001). Reverse sap flow appeared before dawn on July 18th with high air humidity caused by the humidifier. This phenomenon did not occur from the nighttime of July 18th to the early morning of July 19th when the air humidity was low without the humidifier working; small positive sap flows occurred throughout the night. Similar patterns of sap flow reversals were observed on other days with artificial humidification. In addition, reverse flow can happen not only during the more humid nighttime, but also during the daytime under certain rainfall events. Sap flow rate decreased sharply and was even negative in the daytime because of precipitation on July 22nd (Figure 2). No negative flow occurred at midday on July 18th when a precipitation event occurred. This was because the ceiling was not uncovered and this prevented rainfall from hitting the leaves.

Reverse flow was first monitored in twigs of* T. ramosissima* when the RH was >85%. Subsequently it was observed in branches and stems, but it did not always persist throughout the night. It can be concluded from Figure 2 that RH > 90% was more favorable to reverse flow, and the magnitude of reverse flow was affected by air moisture under identical soil moisture conditions. For example, air approached or reached saturation on the early morning of July 20th and 23rd and, correspondingly, the magnitude of reverse flow was most significant at these times.

### 3.3. Water Potential

**Figure 3:**
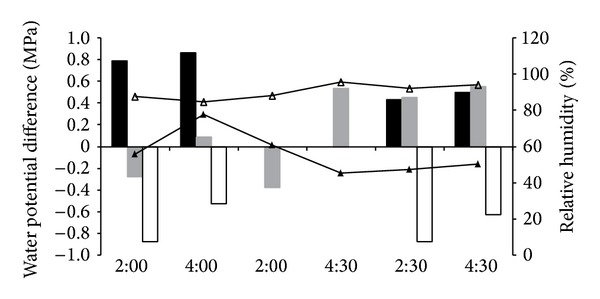
Water potential difference and air relative humidity at specific moments over three days. Black bars show the difference in leaf water potential of* T. ramosissima* between the treatment group and control group; gray bars and nonfilled bars show the water potential differences between leaves and shoots for treatment and control groups, respectively. Line with hollow triangle shows RH in the chamber and line with solid triangle shows RH under natural conditions.

**Figure 4:**
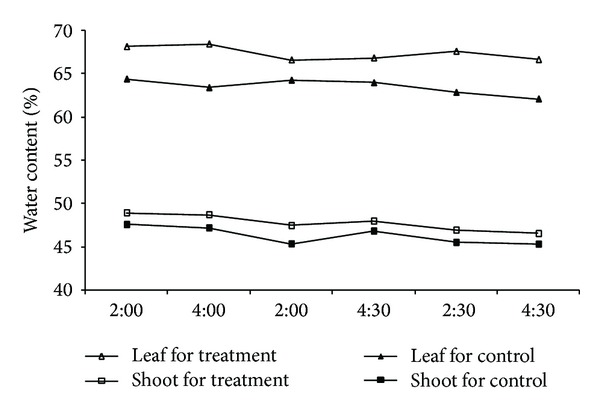
Water contents of leaves and shoots of* T. ramosissima* for control and treatment groups during the same time as mentioned in Figure 3. No error bars are presented for each data point, because the standard error of each data point is significantly smaller than the unit length of water content.

The leaf water potential in the chamber was higher than that under natural conditions (Figure 3, black bars). The water potential difference between leaves and twigs was negative under natural conditions (Figure 3, nonfilled bars), but the difference under high atmospheric humidity was less negative, and even positive (Figure 3, gray bars). This indicated that leaves exposed to high air humidity did take up water directly and improve their water status. Water contents of both leaves and shoots in the chamber were higher than that in the control group (Figure 4). Increases in water contents of the leaves and shoots in the chamber, combined with the reverse sap flow in Figure 2, demonstrated that water taken up by foliage not only partly supplemented leaf water loss, but also was transported to the shoots and branches.

## 4. Discussion

### 4.1. Meteorological Conditions in Favor of Foliar Uptake

**Figure 5:**
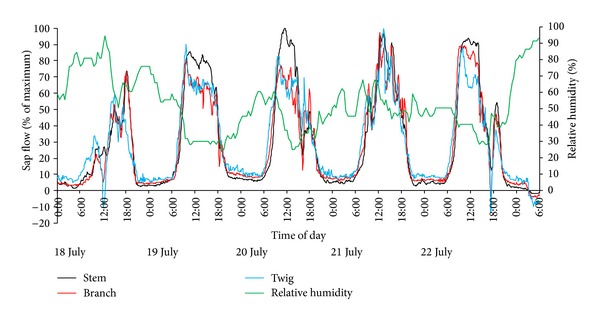
Five days of sap flows at the three different positions on the* T. ramosissima* plant and air relative humidity under natural conditions. Data from the control group synchronize with the treatment group.

Sap flows of* T. ramosissima* were monitored simultaneously under high air humidity conditions and natural conditions. Reverse sap flow was observed in response to RH > 85%, and it was most obvious at RH > 90% (Figure 2). However, positive sap flows almost always occurred throughout the night when RH was low (Figure 5) in the control group. Many studies have shown that foliar water uptake is a prevalent acquisition strategy during the dry season for the coast redwood ecosystem of California where frequent fog events occur [10–12]. Goldsmith et al. [14] monitored reverse sap flows from six different species in the understory of tropical montane and premontane cloud forests and found that the negative velocities corresponded tightly with leaf-wetting events. Besides field experiments, many laboratory experiments have been conducted to study foliar water uptake by controlling the change of environmental parameters. Some data from laboratory work have verified that leaves of plants can absorb water directly when exposed to a prolonged period of simulated fog immersion [9, 15, 26] or a saturated atmosphere [5]. Thus it can be seen that foliar water uptake, occurring in the field or the laboratory, corresponds to very high air humidity. However, foliar water uptake was not inevitably persistent even through maintained high humidity conditions [14]. Moreover, although the RH in the canopy is not very high, sometimes foliar water uptake can still be observed when rainfall occurs. For example, in this study, reverse sap flow was recorded at noon on July 18th (Figure 5), as well as in the afternoon of July 22nd when a thunder shower lasted about 1 h (Figures 2 and 5).

Many studies have demonstrated that leaves can absorb liquid water deposited on leaf surfaces [5, 9, 11–14], but knowledge is lacking about whether leaves absorb water vapor from saturated, or even unsaturated air, though it has been reported that leaves can absorb water vapor directly from a saturated atmosphere [5, 27, 28]. When RH in the chamber reached 85%, a water film formed on the leaf surface as the temperature of the leaf surface was lower than the air temperature. A concrete value of leaf wetness was not measured; it was simply observed. Leaf surfaces in the natural environment were judged as not wet, even when RH was >90% during clear nights. Reverse sap flow was recorded in the early morning of July 23rd for both the treatment and control group (Figures 2 and 5). Given that leaves were not wet in the natural environment with RH > 90%, it was suggested that* T. ramosissima* absorbs not only liquid water from leaf surfaces but also water vapor from a high humidity atmosphere. This requires further substantiation.

### 4.2. Dynamics and Pathways for Foliar Uptake

Water can move into and through plants in both positive and negative directions when the proper gradient is established [20]. The positive water potential difference from the exterior surfaces of the leaf to the interior forms the driving force for foliar water uptake [29]. Air temperature in the chamber at night was nearly always lower than 20°C because of humidification. When RH was 97.97% and temperature was 18.87°C, the atmosphere water potential was −2.78 MPa. The leaf water potential of desert plants in arid regions is generally very low. The leaf water potential of* T. ramosissima* in this study reached −4.78 MPa when the transpiration rate was high at daytime and was near to −6 MPa in some individuals. It was often around −3 MPa even at night. Thus, the positive water potential differences from the atmosphere and leaf surface to leaf interior possibly made conditions favorable for foliar water uptake. Generally, atmosphere water potential was significantly lower than plant water potential. So plants, especially desert plants, should possess some special morphological characters that facilitate uptake.

At present, many studies have identified that water can diffuse into leaves through the cuticle [9, 12, 14], hydathodes [3], and absorbent trichomes [21]. The cuticle is not an impermeable barrier and is capable of transmitting water to the epidermis [21]. Environmental damage can significantly affect the leaf surface permeability [13]; the capacity of foliar uptake can be increased when high wind and precipitation abrade the cuticle [13, 30]. Leaves of* T. ramosissima* are short, bulbous, and scaly, are covered by a cuticle, and have salt glands on the abaxial side and protuberances on the adaxial side. Their green photosynthetic shoots also have micromorphological characters similar to leaves [31]. Blowing sand occurs frequently in the chosen sample plot, so the capacity for foliar uptake by* T. ramosissima* is most likely improved when the leaf cuticular integrity is decreased by the blowing sand. Salt, secreted by the salt glands on the leaves and green photosynthetic shoots, has the ability to absorb water vapor and liquid water. In addition, leaves of* T. ramosissima* have a higher specific surface area. These characteristics benefit the water uptake by leaves and shoots. It has been reported that the bark of twigs can also absorb water [32]. Reverse sap flow was first observed in twigs and then in branches and stems (Figure 2), indicating that water absorbed by leaves or shoots can be transported from branches to stems.

### 4.3. Magnitude and Significance of Foliar Uptake

Foliar uptake improved plant water status distinctly when leaves were submerged in water or exposed to high atmospheric humidity. Both mature and tender leaves exhibited significant water absorption: ~0.462 g g^−1^ and 0.341 g g^−1^, respectively. This indicated that mature leaves of* T. ramosissima* had a greater capacity for foliar uptake than tender leaves. The difference in capacity of foliar uptake between mature and tender leaves was mainly attributable to the different initial leaf water content before submergence. It has been reported that old leaves of* Sequoia sempervirens* absorbed more fog water than young leaves [11]. The mean increase in leaf water content after submergence was near to 30% for mature leaves and a little more than 20% for tender leaves. In contrast, foliar uptake of eight redwood forest species, frequently subject to fog, increased leaf water content by 2–11% [12] and foliar uptake in* Abies fraseri* and* Picea rubens* increased it by 3.7–6.4% [26]. The difference was mainly attributed to the different leaf water potentials and initial water content between the desert plant species and cloud forest species.

Table 2 presents accumulated sap flow values of two stems from the treatment group and the control group under different weather conditions during July 18th to 28th. Accumulated sap flow was normalized by stem cross-sectional area (g cm^−2^). The diameter of the stems from the control group was 22.01 mm and 20.22 mm for the treatment group. The air in the chamber was humidified artificially at night from July 18th to 23rd and July 28th and not humidified on other days. For the treatment group, negative sap flows were recorded when atmospheric humidity was >85%, especially >90%. Moreover, the magnitude of reverse sap flow was affected markedly by the degree of humidification. There are differences in negative sap flow rate among different species. The reversal rates of sap flow in* T. ramosissima* peaked at approximately 10.71% of maximum transpiration rates in our humidification experiment (Figure 2), whereas it peaked at 5–7% in* S. sempervirens* [11] and 25% in Drimys brasiliensis [15]. The causes of the difference between species require further study.

The accumulated amount of reverse sap flow in the stems from the treatment group was 43.21 g (13.47 g cm^−2^) during the night on July 20th, 42.17 g (13.14 g cm^−2^) on July 23rd, and 36.20 g (11.28 g cm^−2^) on July 28th (Table 2). On the three nights when the air was humidified greatly, the magnitude of reverse flow was more significant. According to the crown area of the stem, the reverse sap flow can be expressed in mm; 43.21 g of reverse sap flow was equivalent to 0.18 mm rain being fully absorbed by leaves. Foliar uptake might actually be underestimated by sap flow as much of the water absorbed through leaves probably first refills the plant water storage tissues [11].

**Figure 6:**
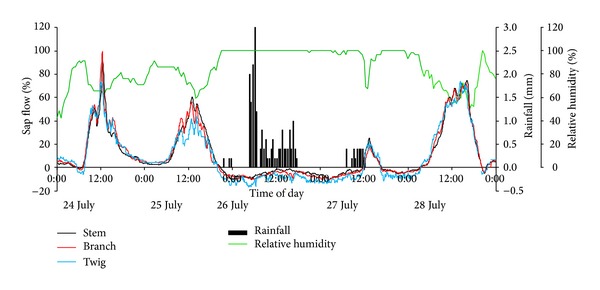
Sap flows at the three different positions on the* T. ramosissima* plant for the control group during continuous rain events.

The study area belongs to an area that typically receives rain and heat over the same period. According to the rainfall data from 2000 to 2013, >72% of rainfall occurs between June and September.  Table 3 presents rainfall events from the automatic weather station over July 2013 in the study area. Of these 12 rainfall events, eight occurred from late afternoon to early morning. This period was favorable for rain droplets to remain on the leaf surfaces for some time because of weak solar radiation. The time interval between the two rainfall events did not exceed 4 days, except one 12-day interval. Small rainfall events (≤5 mm) had the highest frequency. Besides these rainfall events in Table 3, there are several trace rainfall events (<0.2 mm per half hour), which cannot be recorded by the automatic rainfall station. For example, two trace rainfall events occurred on July 20th and 24th during the experimental period in the field. The actual duration was generally longer than the recorded duration because the precipitation was not recorded when its intensity was <0.2 mm per half hour. Small rainfall events were dominant among all the events and their significance cannot be ignored, especially for desert plants. Frequent small rainfall events can effectively suppress transpiration and even lead to negative sap flow to compensate water loss by plants (Figures 5 and 6).

In the early morning of July 24th, as shown in Figure 6, an obvious negative sap flow in the twig was recorded because of a trace rainfall event, but the reverse flow phenomenon was weak in the branch and stem. This also happened in the early morning of July 23rd (Figure 5). When the rainfall events lasted from July 25th to 27th, whether in the daytime or at night, significant and continuous reverse sap flow was recorded (Figure 6) and the magnitude of reverse flow for the control group was much bigger than the treatment group (Table 2). Shortly after the rainfall began on July 25th, reverse flows appeared at the three different positions on the* T. ramosissima* plant and rapidly became greater as the rainfall continued, but the negative sap flow rate did not increase infinitely with rainfall. Reverse sap flow in this study also exhibited some daily variation under 24 h of 95%–100% humidity with a greater reverse flow at night and lower reverse flow at midday. The effect of rainfall on reverse sap flow did not stop immediately after the continuous rainfall events stopped. Negative sap flow was still recorded from nighttime of July 27th to predawn of July 28th, and even weak and temporarily negative flow appeared on the night of July 28th. Leaf water content for the control group was determined before and after rainfall. It was 61.73 ± 0.38% (*n* = 6) at 2 pm on July 24th (before rainfall) and 66.49 ± 0.45% (*n* = 6) at 2 pm on July 27th (after rainfall). The increased leaf water content was similar with the increment before and after leaf submergence in Figure 1(a). The precipitation had an increased trend in the west of the arid zone in China [33], which may be favorable to foliar water uptake.

The influence of foliar uptake on carbon gain is controversial. Some consider that foliar uptake improves carbon gain by reducing leaf water deficit [10, 19], but some have reported that water films on leaf surfaces constrain physiological gas exchange and reduce photosynthetic carbon uptake during leaf wetting [24]. Net photosynthetic rates and transpiration rates for leaves in the treatment group were greater than those in the control group (data not shown), because humidification was only conducted at night, not in a 24 h period. The changes of chlorophyll fluorescence parameters are seldom addressed when a plant is subject to high atmospheric humidity conditions. The specific influences of foliar uptake on photosynthetic and chlorophyll fluorescence parameters will be discussed in a future article.

## 5. Conclusions

This study has shown that the desert species* T. ramosissima* has the capacity for foliar water uptake. The water uptake experiments using submerged leaves yielded an average absorption capacity of 0.462 g g^−1^ for mature leaves and 0.341 g g^−1^ for tender leaves per leaf dry weight. The daily variations of sap flow from the control and treatment groups indicated that high atmospheric humidity and lower leaf water potential can be beneficial to foliar uptake. The surface micromorphologies of leaves and green photosynthetic shoots of* T. ramosissima* are also conducive to capturing liquid water deposited on the surfaces of leaves and shoots as well as water vapor from humid atmospheres. In the chamber, when RH was >85%, especially >90%, reverse sap flow was recorded and its magnitude was closely correlated with the degree of humidification. In natural environments, a small reverse sap flow was also recorded when the RH was >90%. It is very difficult to distinguish the forms of water taken up by leaves in the chamber. In addition, foliar uptake occurs not only during the more humid nighttime, but also during the daytime under certain rainfall events. For the 20.22 mm diameter stem, the percentage of negative sap flow rate accounting for maximum sap flow rate reached 10.71%, and the amount of reverse sap flow at night was equivalent to 0.18 mm rain being fully absorbed by leaves. Water, absorbed by leaves and transported to branches and stems, played an important role in improving the plant water status and physiological processes.

## Figures and Tables

**Table 1:** Precipitation features from July 18th to 22nd, 2013.

Date	Weather	Time of rain	Time of duration (h)	Rainfall (mm)
18th	Shower, cloudy	Around midday	2.5	0.2~0.4
20th	Mostly clear	The afternoon	1	0.2
22nd	Mostly clear	The afternoon	1	0.2~0.4

**Table 2:** Accumulated sap flow of *T.  ramosissima* for treatment and control groups during the daytime and nighttime under different weather conditions in July.

Weather	Date	Sap flow of treatment group (g cm^−2^)				Sap flow of control group (g cm^−2^)			
		Daytime		Nighttime		Daytime		Nighttime	
		Positive	Negative	Positive	Negative	Positive	Negative	Positive	Negative
Shower, cloudy	18th	90.27	0.00	9.53	1.20	83.63	0.00	11.82	0.00
Sunny	19th	203.96	0.00	18.85	1.97	162.07	0.00	16.19	0.00
Mostly clear	20th	196.87	0.00	11.65	13.47	152.25	0.00	18.95	0.00
Mostly clear	21st	185.21	0.00	10.84	0.37	149.73	0.00	15.38	0.00
Mostly clear	22nd	210.09	0.00	9.96	2.97	156.57	0.00	15.46	0.00
Sunny	23rd	210.64	0.00	10.70	13.14	170.12	0.00	7.09	0.80
Cloudy	24th	141.85	0.00	11.11	0.12	83.99	0.00	9.53	0.12
Rainy	25th	114.78	0.00	9.79	3.86	84.48	0.00	5.57	3.91
Rainy	26th	0.00	10.8	0.00	17.04	0.00	8.59	0.00	16.26
Rainy	27th	46.48	2.44	1.42	11.06	12.51	3.69	0.00	14.19
Cloudy	28th	175.14	0.00	6.50	11.28	121.22	0.00	6.79	2.06

Note: the daytime is from 7 am to 7:30 pm; the rest of the time is the nighttime.

**Table 3:** Rainfall profile of the study area in July 2013.

Date	Start time	Rainfall (mm)	Duration (h)	NRDBRE (d)	NRDARE (d)
2013.7.3	3 pm	2	3.5	12	3
2013.7.7	12 pm	3	6	3	0
2013.7.8	1:30 am	1.8	4	0	0
2013.7.9	3 am	16	12.5	0	1
2013.7.11	5 pm	0.2	0.5	1	2
2013.7.14	5:30 pm	0.6	1.5	2	3
2013.7.18	12:30 pm	0.2	0.5	3	3
2013.7.22	5 pm	0.2	0.5	3	2
2013.7.25	8:30 pm	0.6	1.5	2	0
2013.7.26	3:30 am	19.4	12	0	0
2013.7.27	6 am	3	5.5	0	3
2013.7.31	2:30 pm	3.4	3	3	1

Note: NRDBRE: no rain days before rainfall events; NRDARE: no rain days after rainfall events.
